# Botulinum Toxin Type A Therapy in Migraine: Preclinical and Clinical Trials

**DOI:** 10.5812/ircmj.7704

**Published:** 2013-10-05

**Authors:** Yu-Feng Shao, Yi Zhang, Peng Zhao, Wen-Jun Yan, Xiang-Pan Kong, Lin-Lan Fan, Yi-Ping Hou

**Affiliations:** 1 Department of Neuroscience, Anatomy, Histology and Embryology, School of Basic Medical Sciences, Lanzhou University, Lanzhou, China; 2 Department of Neurology and Pain Treatment, Gansu Provincial People Hospital, Lanzhou, China; 3 Gansu University of Traditional Chinese Medicine, Lanzhou, China; 4 Medical Experimental Center, School of Basic Medical Sciences, Lanzhou University, Lanzhou, China

**Keywords:** Botulinum Toxin Type A, Nitroglycerin, Randomized Controlled Trial

## Abstract

**Background:**

Botulinum toxin type A (BTX-A) has been reported to be effective for the therapy for migraine. The purpose of this study was to investigate the effect of BTX-A on the immunoreactive levels of calcitonin gene-related peptide (CGRP) and substance P (SP) in the jugular plasma and medulla oblongata of migraine in rats induced by nitroglycerin (NTG), and then to evaluate and compare the effectiveness of fixed (muscle)-sites and acupoint-sites injection of BTX-A for migraine therapy of patients in a randomly controlled trial extending over four months.

**Materials and Methods:**

Rats with NTG-induced migraine were subcutaneously injected with vehicle or BTX-A (5 U/kg or 10 U/kg bodyweight). CGRP- and SP-like immunoreactivity (CGRP-LI and SP-LI) were determined by radioimmunoassay. In clinical trials, sixty patients respectively received BTX-A (2.5 U each site, 25 U per patient) at fixed-sites (group F, n = 30) including occipitofrontalis, corrugator supercili, temporalis and trapezius or at acupoint-sites (group A, n = 30) including EX-HN3, EX-HN5, GV20, GB8, GB20 and BL10.

**Results:**

Local BTX-A injection suppressed NTG-induced CGRP-LI and SP-LI levels in jugular plasma and oblongata. BTX-A injection for both groups with migraine significantly reduced the attack frequency, intensity, duration and associated symptoms. The efficacy of BTX-A for migraine in group A (93% improvement) was more significant than that in group F (83% improvement) (P < 0.01).

**Conclusions:**

The evidence that BTX-A decreases NTG-induced CGRP-LI and SP-LI levels in trigeminovascular system suggests that BTX-A attenuates migraine by suppression of neuropeptide release. BTX-A injections for migraine at acupoint-sites and fixed-sites are effective. Acupoint-sites BTX-A administration shows more efficacy for migraine than fixed-sites application.

## 1. Background

Migraine is characterized as a neurovascular disorder of recurring, throbbing headache, and often associated with aura, nausea, vomiting, photophobia, phonophobia, fatigue and enhanced irritability (1). Both pericranial muscle tenderness (muscle allodynia) and also cutaneous allodynia (scalp allodynia) have been described during migraine attacks (2, 3). Nociceptive inputs of myofascial origin have been postulated to play a role in migraine pathogenesis (4).

Migraine is a complex, neurovascular disorder in which genetic and environmental factors interact (5). One theory, based on preclinical observations, is by activation of trigeminal sensory fibers which lead to a painful neurogenic inflammation within the meningeal vasculature mediated by neuropeptide release from the trigeminal sensory fibers and is characterized by plasma protein extravasation, vasodilation, and mast cell degranulation (6). Previous experiments have demonstrated that unmyelinated nociceptive trigeminal fibers provide the major source of sensory innervation for cranial blood vessels (trigeminovascular system) (7) and contain neuropeptides such as calcitonin gene-related peptide (CGRP) and substance P (SP) (7). These neurons convey nociceptive impulses to the brain and at the same time may co-release CGRP and SP from the peripheral endings, thus evoking a variety of effects collectively known as neurogenic inflammation (8). The hypothesis that the activation, for unknown reasons, of peptide-containing trigeminal neurons leads to the generation of pain and simultaneously to inflammation effects in cranial blood vessels has the advantage of offering a unifying explanation for the crucial symptoms of migraine, such as pain and vasodilation (8).

Botulinum toxin type A (BTX-A) is a focally administered neurotoxin, which inhibits the release of acetylcholine at the neuromuscular junction and is used therapeutically in disorders characterized by muscle hyperactivity (9). It has also been reported to be effective in the therapy of migraine (10-12) or chronic migraine (13, 14). Until now, most researchers have based reports of improvements in the symptoms of migraineurs with BTX-A injections on studies in which fixed-sites in the forehead, temple and neck muscles are injected for muscle-relaxation (10, 12, 15). New evidences have recently demonstrated that acupoint-sites injections are useful in alleviating or eliminating symptoms of migraine (16, 17). In traditional, Chinese medicine acupuncture is considered as one of the most effective treatments for migraine (18, 19). Acupoints are believed to correspond to energy channels that circulate through the body and connect the organs and the viscera (20). Acupoint injection as a therapeutic technique is well documented and has been indicated to have more quick and powerful clinical effects than muscle and subcutaneous injection (21) and has been used to treat some diseases including headache, (22) myofascial pain, (23) temporomandibular disorder (24) and rheumatoid arthritis, (25) although the mechanism of acupoint injection is unclear.

## 2. Objectives

The purpose of the present study was to investigate the effect of BTX-A on CGRP and SP levels in the jugular plasma and medulla oblongata containing caudal trigeminal nucleus of the rat model of migraine induced by nitroglycerin (NTG), and then to determine, in a randomized controlled trail, whether acupoint-sites injection of BTX-A is effective in the treatment of migraine and to compare its efficacy with that obtained by fixed-sites injection of BTX-A, which is one of the most commonly used methods of BTX-A for migraine therapy in patients.

## 3. Materials and Methods

### 3.1. Experimental Trial

#### 3.1.1. Animals

Pathogen-free adult female Sprague-Dawley (SD) rats (Experimental Animal Center, Lanzhou University, China) weighing 250-300 g were housed individually in cages. The animals were raised and maintained under standard laboratory conditions with a 12-hour light-dark cycle (light on 07:00-19:00 h). Food and water were offered ad libitum. Every effort was made to minimize the numbers and any suffering of the animals used in the following experiments. All the protocols in this study followed the guidelines of the International Association for the Study of Pain and the European Communities Council (86/609/ECC) and were approved by the Institutional Animal Care and Use Committees of Gansu Province Medical Animal Center and Lanzhou University.

#### 3.1.2. Model of Migraine Induced by NTG and BTX-A Administration 

According to Vamos et al. (26) briefly, rats received frontal and temporal subcutaneous (s. c.) injection of NTG (10 mg/kg body weight, H14022197, Kangbao Bio-products Co. Ltd., Shanxi, China) to produce NTG-induced migraine. The doses of BTX-A (GMP Nos. Co868, S10970037, 2008101, Lanzhou Institute of Biologic Products, Lanzhou, China) were based on an earlier work (27, 28); prepared freshly with 0.9% NaCl vehicle in 0.1 ml of solution containing 5 U or 10 U BTX-A, and kept at 4°C and used within 4 h. For radioimmunoassay, the animals were divided into four groups (n = 8, per group): control group in which animals were without any treatment; NTG + vehicle, NTG + BTX-A 5 U and NTG + BTX-A 10 U groups, in all of which animals respectively received a vehicle or BTX-A (5 U/kg or 10 U/kg bodyweight) s.c. injection at the frontal and temporal area two hours after NTG administration. 

#### 3.1.3. Sample Preparation and Radioimmunoassays

Animals were beheaded 24 hours after vehicle or BTX-A administration for 2 ml blood withdrawn and medulla oblongata containing caudal trigeminal nucleus harvest. Sample preparation and radioimmunoassay (RIA) were performed as described in our previous studies (28). Briefly, tubes containing the blood samples were immediately transferred into a 4°C bath. Medulla oblongata was sequentially weighed, rinsed with 0.9% NaCl, dried with filter paper, grinded in 1 ml of 4% acetic acid and boiled (95°C) for 3 min in 0.9% NaCl containing 0.5% bovine serum albumin (BSA). Blood and homogenate from oblongata were sequentially centrifuged at 4°C for 15 min, and plasma and supernatant fractions were aliquoted and stored at -70°C until extraction. Extraction was carried out in Sep-Pak C18 (Millipore, MA) cartridges activated with 5 ml of methanol and 10 ml of H_2_O. Cartridges were then loaded with plasma to which 0.1 ml/ml of 1 N HCl had previously been added. Samples were washed with 10 ml of H_2_O followed by 10 ml of H_2_O with 4% acetic acid. Peptides were eluted with a 5 ml solution containing 90% methanol and 10% acetic acid. Samples were dried under a nitrogen stream, and after reconstitution with 0.1 M phosphate buffer (pH 7.4), were stored at -70°C until assayed. Rabbit anti-CGRP and SP RIA kits (Navy Radioimmunoassay Technique Center, Beijing, China) were carried out according to the procedure reported earlier (28) for determining CGRP- and SP-like immune-reactivity (CGRP-LI and SP-LI), respectively. The assay was performed as recommended by the supplier, and the gamma radioactivity in the remaining pellet was counted (Radio-immunoassay Gamma Photon Counter, Xian, China).

### 3.2. Clinical Trial

This clinical trial was performed on patients with migraine admitted to the Department of Neurology and Pain Treatment, Gansu Province People Hospital, China. According to a predetermined computer-made randomization list, the eligible patients were assigned to fixed (muscle)-sites injection of BTX-A (group F, n = 30) or to acupoint-sites injection of BTX-A (group A, n = 30). The patients had an equal probability of being assigned to either treatment groups. Each patient was asked, before enrollment, to give an informed consent to participate in the study. For one month prior to and four months following treatment, subjects recorded the following headache parameters that contained 56 item in diaries provided by the investigators: occurrence of migraine and nonmigraine headaches, the start and stop time of migraine, migraine severity, the presence of migraine aura, migraine associated symptoms, and acute migraine medications or treatment used. This study was conducted in compliance with the institutional review broad regulation, informed consent regulations, the Declaration of Helsinki, and the International Headache Society (IHS) guidelines 2004 (29).

#### 3.2.1. Subjects

Patients of both genders (21 men and 39 women), aged 19 to 49 years, who had a history of IHS-defined migraine for one to 16 years with or without aura, were selected for this study. Patients who experienced medical or neurological conditions likely to induce migraine were considered ineligible for this study.

#### 3.2.2. Treatment

**Figure 1.**
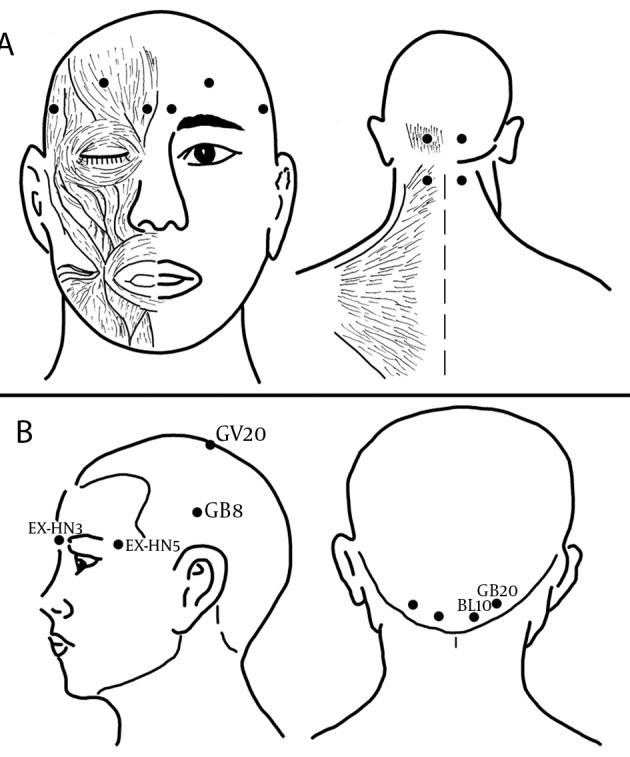
Sites of BTX-A Injection Used for Migraine Treatment in This Study (A) In group F, these sites (●) are respectively located at the frontal and occipital belly of occipitofrontalis, corrugator supercilii, temporalis and superior part of trapeziue muscle. (B) In group A, these sites (●) are EX-HN3 (Yintang), at the midpoint of the line connecting the two medial ends of eyebrows; EX-HN5 (Taiyang), at the point of intersection of the continuations of the eyebrow and the lower eyelid in the lateral direction, on the lateral border of the orbita; GV20 (Baihui), at the middle of the vertex, on the line connecting the apexes of the two ears; GB8 (Shuaigu), directly above the ear apex, 1.5 inches above the hairline; GB20 (Fengchi), at the posterior lateral aspect of the neck, in the fossa between the superior margins of the trapezius and sternocleidomastoid muscles; and BL10 (Tianzhu), 1.3 inches lateral to the point located 0.5 inches directly above the midpoint of the posterior hairline, in the depression lateral to the border of the trapezius muscle.

The application sites of group F were respectively at frontal and occipital belly of occipitofrontalis, corrugator supercili, temporalis and superior part of trapeziue (Figure. 1A), which have been mostly selected to treat migraine with BTX-A in previous studies (10, 11, 15) (30, 31). The injection sites in group A were respectively at EX-HN3 (Yintang), EX-HN5 (Taiyang), GV20 (Baihui), GB8 (Shuaigu), GB20 (Fengchi) and BL10 (Tianzhu), based on predetermined and well-known Chinese acupoints for the treatment of migraine (Figure. 1B) (32). When patients in both groups finished the prescribed diary of the run-in period, they were administered BTX-A through a 1-inch, 30-gauge needle into each site 0.1 ml of saline containing BTX-A 2.5 U for a total dose of 25 U per patient. All of the injections were respectively performed by two of the authors (Y. WJ and Z. Y) to assure consistency. 

#### 3.2.3. Outcome Measure

Operators who did not know the very group of each patient conducted the analysis of the diary data. All patients in group F and A underwent a control visit by these operators every month. Evaluation of different parameters was carried out monthly such that; (I) frequency was recorded as the number of migraine attacks per month; (II) intensity of migraine was measured on a visual analogue scale (VAS) ranging from 0 to 10: 0, no pain; 10, unbearable pain; 1-3, mild; 4-6 moderate; 7-9, severe pain; (III) duration of each attack; (IV) migraine associated symptoms with scale ranging from 0 to 3: 0, lack; 3, three to four; 2, two; 1, one of symptoms including vomiting, nausea, phonophobia and photophobia. Their variations with respect to the run-in period (1 month prior to treatment, time 0 = T0) were calculated every month (1 month = T1; 2 months = T2; 3 months = T3; 4 months = T4) as outcome measure.

### 3.3. Statistical Analyses

Data in the experiments were expressed as means ± SEM and analyzed using one-way ANOVA followed by least significant difference (LSD) test for the variation of CGRP-LI and SP-LI in four groups. The data in the clinical trial were analyzed by one of the authors (S. YF) who did not participate in group division and treatment. The values were presented as means ± SEM. The quantitative data were analyzed with one-way ANOVA followed by LSD test with SPSS 11.5 for Windows. For binomial qualitative data, comparisons between treatment groups were done with chi-square tests. Differences between means were considered significant at P < 0.05.

## 4. Results

### 4.1. Preclinical Trial

#### 4.1.1. Effect of BTX-A on NTG-induced CGRP- and SP-LI Levels in Jugular Plasma and Oblongata

**Figure 2.**
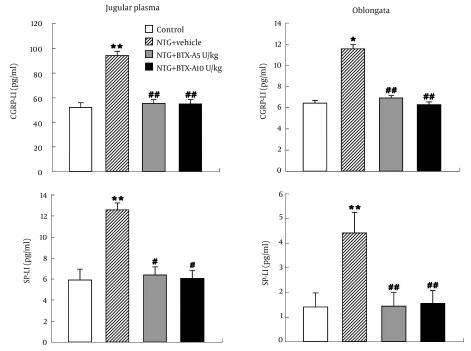
The CGRP- and SP-LI Levels in Jugular Plasma and Oblongata of Rats Detected by RIA in Control, NTG + Vehicle, NTG + BTX-A 5 U/kg and NTG + BTX-A 10 U/kg Group Respectively Higher levels of CGRP-LI (upper panels) and SP-LI (lower panels) were respectively detected in jugular plasma and oblongata following NTG + vehicle treatment. BTX-A administration decreased NTG-induced CGRP- and SP-LI levels in jugular plasma and oblongata. The effect of BTX-A 10 U/kg on NTG-induced CGRP- and SP-LI levels was not different from that of BTX-A 5 U/kg. *P < 0.05, **P < 0.01, vs. control; #P < 0.05, ##P < 0.01, NTG + BTX-A 5 U/kg or NTG + BTX-A 10 U/kg group vs. NTG + vehicle group.

NTG s.c. injection into frontal and temporal area of rats induced a marked increase of CGRP-LI levels in jugular plasma (1.8-fold, P < 0.01) and oblongata (1.8-fold, P < 0.05) compared with control (Figure 2, upper panels). Local administration of BTX-A two hours after NTG treatment, decreased CGRP-LI levels in jugular plasma (P < 0.01) and oblongata (P < 0.01) than vehicle administration did (Figure 2, upper panels). However, the inhibitory effect of BTX-A 10 U/kg on CGRP-LI levels was not different from that of BTX-A 5 U/kg (P > 0.05). 

In comparison with the control, NTG induced a significant increase in SP-LI levels in jugular plasma (2.14-fold, P < 0.01) and oblongata (3.14-fold, P < 0.01). Local BTX-A s.c. injection two hours after NTG treatment respectively suppressed SP-LI levels in jugular plasma (P < 0.05) and oblongata (P < 0.01) compared with vehicle administration (Figure 2, lower panels). Differences between BTX-A 5 U and 10 U/kg on SP-LI levels were not found. 

### 4.2. Clinical Trial

#### 4.2.1. Subjects

All participants in this trial were Chinese. The baseline characteristics including age, gender, weight and the duration of migraine history in the two groups were not significantly different (Table 1). There were no statistically significant differences between group A and group F in migraine severity, distribution (unilateral versus bilateral), type of pain, or effect of physical activity before treatment. 

**Table 1.** Subjects Characteristics

Group	Case (n)	Gender M/F (n)	Age (y)	Weight (kg)	Stature (cm)	Duration of Migraine (y)
**F**	30	10/20	40.2 ± 0.7	60.2 ± 0.6	163.1 ± 0.4	5.1 ± 0.8
**A**	30	11/19	40.3 ± 0.9	61.1 ± 0.3	164.2 ± 0.5	6.3 ± 0.5

#### 4.2.2. Effect of BTX-A Injection in Both Groups on the Attack Frequency, Intensity, Duration and Associated Symptoms of Migraine

**Figure 3.**
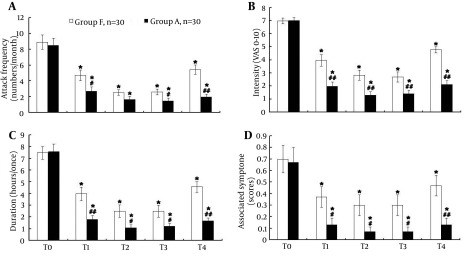
The attack Frequency, Intensity, Duration and Associated Symptoms of Migraine During the run-in Period (T0) and After 1 Month (T1), 2 Months (T2), 3 Months (T3), and 4 Months (T4) Therapy in Group F and Group A BTX-A injection in both groups induced a significant reduction in attack frequency (A), intensity (B), duration (C) and associated symptoms (D). The reduction in attack frequency (A), intensity (B), duration (C) and associated symptoms (D) in group A was significantly more different than that in group F. *P < 0.01 vs. T0; #P < 0.05, ##P < 0.01 vs. group F.

BTX-A injection in both groups induced a significant decrease in attack frequency of migraine for four months compared with the run-in period (P < 0.01; Figure 3A). However, the reduction of attack frequency in group A was greater at T4 (P < 0.01; Figure 3A), and significant at T1 and T3 (P < 0.05; Figure 3A) compared with group F. 

As shown in Figure 3B, the reduction in migraine intensity was always present in both groups after treatment (from T1 to T4) compared with the run-in period (T0) (P < 0.01; Figure 3B). In addition, the reduction of migraine intensity in group A was statistically different from group F from T1 to T4 (P < 0.01; Figure 3B). 

The mean duration of each attack after BTX-A treatment in both groups was significantly reduced compared to that before the treatment (P < 0.01; Figure 3C). Comparison between the two groups, revealed that the decrease in duration of each attack in group A was greater at T1 and T4 (P < 0.01; Figure 3C), and more significant at T2 and T3 (P < 0.05; Figure 3C). Most participants during the run-in period experienced at least one of the four symptoms typically associated with a migraine attack (i.e. vomiting, nausea, photophobia, phonophobia). The decrease of migraine associated symptoms in both groups after BTX-A treatment was significant for four months compared with the run-in period (P < 0.01; Figure 3D). Comparison of the two groups revealed that the reduction of migraine associated symptoms in group A was statistically different from group F, respectively, from T1 to T3 (P < 0.05) and at T4 (P < 0.01; Figure 3D). 

#### 4.2.3. Efficiency of Different Sites Injection

Following BTX-A injection in group F, 25 (83%) of 30 patients noted improvement in migraine, in which 6 (20%) were attack-free, 10 (33%) were markedly effective, 9 (30%) were effective and 5 (17%) were invalid. Whereas, in group A of BTX-A injection, 28 (93%) of 30 patients noted improvement in migraine, in which 10 (33%) were attack-free, 14 (47%) were markedly effective, 4 (13%) were effective and 2 (7%) were invalid. Data from this study demonstrated that BTX-A injection in group A for migraine treatment was more efficient than in group F (P < 0.01).

#### 4.2.4. Side Effects

Two cases (7%) from 30 patients in group F after BTX-A injection appeared transient unilateral upper eyelid ptosis that lasted for one week for one patient and two weeks for the other patient. One patient in group F after injection noted an acute pain in the injection sites that disappeared after one night. These three patients were among cases of improvement in group F. One patient in group A after injection felt an ethereal pain on the local skin that lasted for four days, and BTX-A treatment was invalid for this patient.

## 5. Discussion 

In our preclinical trial, frontal and temporal s. c. injection of NTG in rats induced a marked increase of CGRP- and SP-LI levels in jugular plasma and oblongata. The model of migraine induced by NTG, a nitric oxide (NO) donor, has been widely used in animals (26, 33) or humans (34, 35). NO is likely to play a pivotal role in the pathogenesis of migraine, a disorder characterized by pain sensitization associated with cranial vascular changes (36). Therefore, NTG-induced migraine attack appears to be a right model for assessing changes in vasoactive neuropeptides as CGRP and SP which are likely to occur in a spontaneous crisis. Peptide levels obtained in vessel oblongata are so close to the trigeminovascular system that could be considered as reliable predictors of migraine attack. In fact, an increase in immunoreactivity for CGRP has been reported in human blood from the external jugular vein after NTG administration (8).

Two hours after NTG treatment, BTX-A administration decreased CGRP- and SP-LI levels in jugular plasma and oblongata. The mechanism involved is the inhibition of acetylcholine exocytosis through cleavage of synaptosome-associated protein (SNAP)-25, one of the SNRE proteins. A growing amount of evidence reveals that BTX-A also inhibits the release of selected neuropeptide transmitters such as SP and CGRP from trigeminal nerve ends and primary sensory neurons (27, 37) and from autonomic and enteric nervous terminals (28, 38). The exact mechanism of BTX-A-mediated inhibition of SP and CGRP release is unknown. Previous investigations show that BTX-A-induced inhibition of sensory neurotransmitter releases from the trigeminal and dorsal root ganglion neurons is associated with a concentration-dependent cleavage of synaptosome-associated protein SNAP-25 (37). These findings suggest the possibility that the sensory-specific effect of BTX-A might occur through inhibition of sensory neuropeptide vesicle release via a SNAP-mediated mechanism, similar to the blockade of acetylcholine release in motor neurons. This mechanism has been thought to underlie the frequently reported reduction of pain with BTX-A in the treatment of migraine. In our clinical trials, injection of total dose of BTX-A, 25 U, into fixed-sites (group F) or acupoint-sites (group A) could significantly reduce migraine frequency, density, duration and associated symptoms. The beneficial effects of BTX-A were observed mainly from two to four months post-injection, as previously reported for other preventive migraine treatments (6). Improvement produced by BTX-A continues through four months suggesting that the effect of BTX-A on migraine could be beyond four months. The 25-U dose of BTX-A for migraine treatment in our study was proved as an efficacious dosage. Our results were in agreement with the investigative conclusion of Siberstein et al. that 25-U dose of BTX-A was safe and reduced migraine frequency, severity, acute medication use, and migraine-associated symptoms (10).

The main objective in the clinical trial was to compare the efficacy in group F and group A injected with the same dose of BTX-A for migraine treatment. The results of migraine improvement were respectively 93% in group A and 83% in group F. BTX-A-induced reduction of migraine frequency, density, duration and associated symptoms in group A were more significant than that in group F (Figure 3). These evidence suggest that acupoint-site injection of BTX-A is efficient for migraine treatment, and seems better than fixed-sites injection. Acupoint injection of a small amount of drug, vitamin, saline, or plant extract is a recent innovation of traditional acupuncture that aims to enhance and prolong the effect of stimulation of acupoint (39). One suggested advantage of this technique is that it offers the opportunity to standardize and replicate treatment, which is difficult to achieve with classic acupuncture (21). The evidences of most subjects who reported a more powerful stimulation response with acupoint injection may indicate that point injection provides a more powerful clinical outcome, with stronger sensations translated into a stronger clinical response, as outlined by Wang et al., (39) and Luo and Chen (40). Vernejoul and his colleagues have studied the migration of radioactive tracers injected at acupoint-sites using a scintillation camera coupled to a computer system with image analysis capability (41). They found the migration of radioactive tracer from one acupoint to another, along pathways superimposable with the traditional Chinese medicine, taking into account the migration speed and few patterns, does not indicate an intravascular lymphatic origin. These pathways are likely related to connective tissue diffusion following the vascular nervous packs. A rapid and simultaneous response to the acupoint stimulation suggests a neuro-chemical mechanism in information transmission. Several patients in two groups of this study injected with BTX-A had transient unilateral upper eyelid ptosis and injection site pain and recovered quickly without any treatment, confirming the tolerability of BTX-A as reported by other studies (31). Other agents used in the prophylaxis of migraine cause side effects such as fatigue, dizziness, reduced concentration, loss of appetite, weight gain, hair loss, and changes in libido (42). However, BTX-A has not been reported to bring such side effects. In conclusion, the inhibitory effect of BTX-A on NO donor NTG-induced CGRP-LI and SP-LI levels in trigeminovascular system demonstrated in our experimental studies suggests that the effect of BTX-A on migraine is via suppression of neuropeptide release, which plays a pivotal role in neurogenic inflammation of attack. Acupoint-sites and fixed-sites injection of BTX-A for migraine were able to significantly reduce attack frequency, intensity, duration and associated symptoms. Acupoint-sites administration of BTX-A was proved to be more efficient for migraine than fixed-sites application, and thus is a potential method in clinical practices. 
